# Migration, Integration, Survival, and Differentiation of Stem Cell-Derived Neural Progenitors in the Retina in a Pharmacological Model of Retinal Degeneration

**DOI:** 10.1155/2013/752161

**Published:** 2013-02-26

**Authors:** Gustavo Castro, Eduardo Navajas, Michel Eid Farah, Mauricio Maia, Eduardo Buchele Rodrigues

**Affiliations:** Vision Institute, Department of Ophthalmology, Federal University of São Paulo, 04021-001 São Paulo, SP, Brazil

## Abstract

*Purpose*. The purpose of this work was to evaluate the retinal integration and differentiation of neurospheres formed by stem cells and mouse neural progenitor cells injected intravitreally in mice eyes with retinal injury. *Methods*. Eight male C57BL mice, 8 weeks old, were submitted to intraperitoneal injection of sodium iodate (2% NaIO3, 50 mg/kg). After 72 hours, 2 **μ**L of solution with mNPC were injected intravitreally (100.000 cells/**μ**L). After 7 days, their eyes were dissected and cryoprotected in 30% sucrose in PB for at least 24 hours at 4°C. The material was analyzed by immunohistochemistry and the following primary antibodies evaluation. *Results*. The results showed that the grafted cells integrated and survived in the adult mice within the sinner retinal tissue for at least 7 days. Immunohistochemical analysis revealed mature neuronal pattern in some regions. The mNPC population in the transplants was tightly surrounded by neuroretinal cells, suggesting their active role in neuron survival. Notably, the appearance of GFP-positive mNPC was not the result of fusion between donor cells and endogenous neuroretinal cells. *Conclusions*. Migration, survival, and differentiation of mNPCs were observed after 7 days following a single application with neurosphere method. The results may be clinically relevant for future stem cell therapy to restore retinal degeneration.

## 1. Introduction

 Retinal degenerative diseases remain an important cause of blindness in current years. In those conditions retinal cells suffer progressive damage, which cannot be avoided or replaced by modern therapeutic approaches [1]. Cell-based therapies including neural and stem cells represent an excellent perspective for therapy of retinal degenerations by replacing degenerating or lost photoreceptors [2]. The purpose of this study was to evaluate the potential for migration, incorporation, survival, and differentiation of neurospheres containing murine neural progenitor cells (mNPC) and stem cells in a pharmacological model of RPE-degeneration in the mice.

## 2. Materials and Methods

 The work was conducted after approval by the Ethics Committee of Universidade Federal de São Paulo (UNIFESP). For harvesting and culturing of progenitor cells, mouse neural progenitor cells (mNPC) were obtained from E14 (embryonic day 14) C57BL/6-GFP (green fluorescent protein) (Center for Development of Animal Models for Medicine and Biology (CEDEME), Sao Paulo, Brazil) mouse embryos. The male fetuses were placed in a Petri dish containing PBS/2% glucose, and the dissection is made under magnifying lens. The brains were sectioned, and the tissue was incubated with Trypsin-EDTA solution (Gibco, 15400-054) for 15 min at 37°C. Trypsin was inactivated with fetal bovine serum, and, after cell sedimentation, the supernatant was removed, and the cells dissociated in 70% DMEM (Gibco 11965-118), 30% F12 (Gibco 11765-062), 1% PSA (Gibco 15240-062), 2% B27 (Gibco 17504-044), 20 ng/mL EGF (Sigma E9644), 20 ng/mL FGF-2 (R&D 233-FB), and 5 *μ*g/mL heparin (Sigma H3149 100KU). 

**Figure 1:**
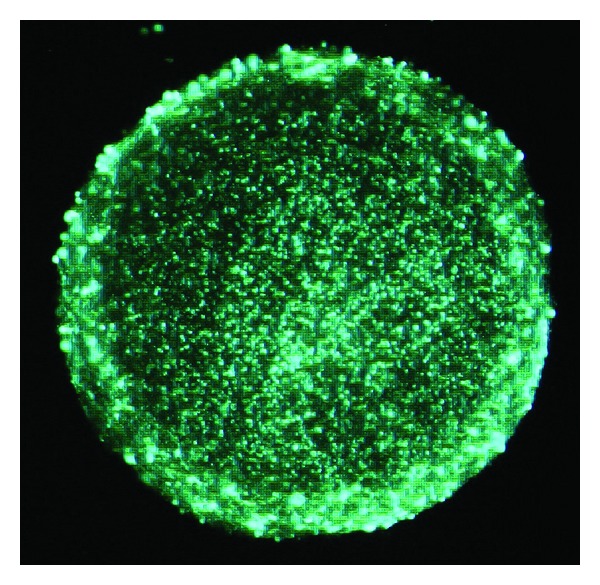
Neural progenitor cells. Formation of neurospheres containing embryonic neural progenitor cells obtaining at E14 that have aggregated in suspension after 10 days in culture. Neurospheres present as non-adherent spherical clusters of cells, they are formed by about 95% neural progenitor cells and 5% stem cells. Many factors contribute to the cellular composition of the neurosphere, key variables including the age of the animal, plating density, culturing techniques, and passage number. 100x.

 The cell suspension was counted in a hemocytometer, and the cells are seeded in a T25 flask at a density equivalent to 100,000 cells/mL. The plating of neurospheres was performed by coverslips coated with poly-l-lysine solution (Sigma P5899, 0,1 mg/mL in milliQ water). Cells were maintained in an incubator at 37°C under a 5% CO_2_ atmosphere. The spheres were transferred to conical tubes and washed carefully 3 times with 8 mL prewarmed DMEM. The spheres were centrifuged at 900 rpm, mixed with growth factors-free medium (DMEM/F12/B27) and kept in those conditions in suspension for 10 days. Every 4 days, half of the volume was replaced with fresh medium. Cells were fixed in 4% PFA, washed in PBS 3 times, and then blocked/permeabilized in 5% goat serum/0.1% Triton X-100 for 30 min (Figure 1).

The animal model for retinal degeneration used was the C57BL/6 (Center for Development of Animal Models for Medicine and Biology (CEDEME)). Eight male C57BL mice, 8 weeks old, were submitted to intraperitoneal injection of sodium iodate (2% NaIO3, 50 mg/kg.). After 72 hours, 2 *μ*L of solution with mNPC was injected intravitreally (100.000 cells/*μ*L). After 7 days, their eyes were dissected and cryoprotected in 30% sucrose in PB for at least 24 hours at 4°C. 

 The material was analyzed by immunohistochemistry, and the following primary antibodies were used: anti *β*-tubulin III (Sigma, USA; mouse IgG2b, 1 : 200), anti-Glial fibrillary acid protein conjugated to fluorescein (GFP; Molecular Probes, Invitrogen, USA; 1 : 300), and anti-DAP (death-associated protein, Sigma, USA; 1 : 50). Primary antibodies were added, and the cells are incubated overnight at 4°C. Cells were washed in PBS. Eyes were perfused with PFA 4% sacarose solution, and retinas were sectioned with cryostat (10 *μ*m thickness). Retinal tissues were analyzed under a fluorescence microscope. The sectioned tissues were mounted over a glass lamina silanized (Superfrost slides, Fisher Scientific) and analysed in confocal microscope (Zeiss, Axiovert 100 M, LSM 510 software).

## 3. Results

**Figure 2:**
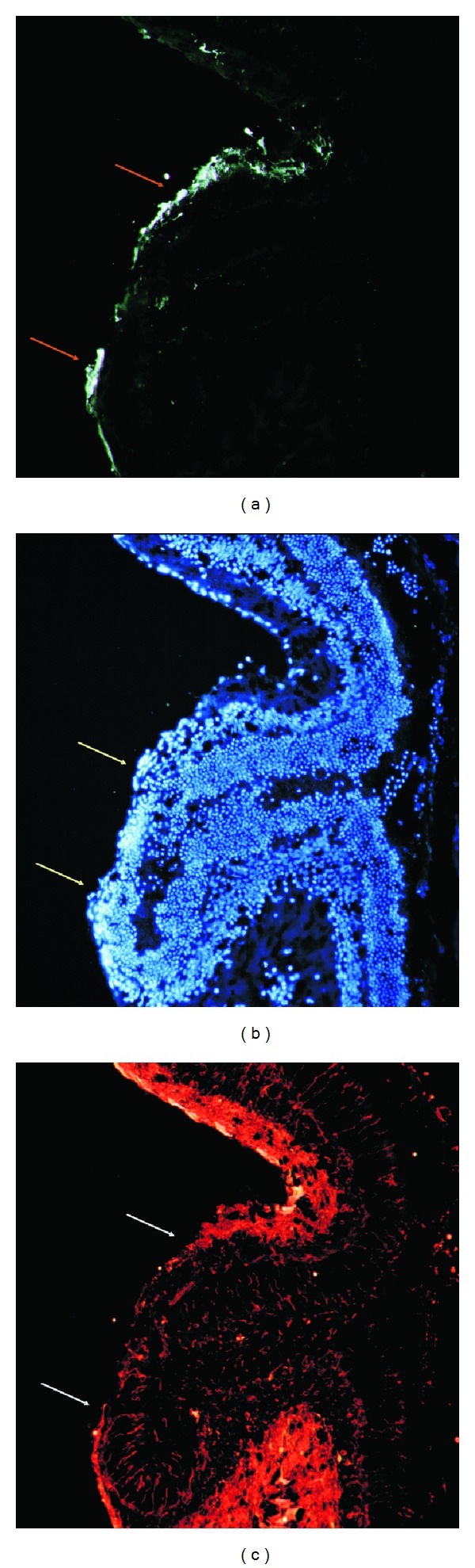
Photograph of a mice retina after immunohistochemistry with three different markers. (a) Following transplantation, GFP-expression allowed histological visualization of integrated cells and extension to superficial retinal layers after 7 days following a single application with neurosphere method. The cells transplanted of the mNPC into the vitreous cavity survived, migrated, and became located into the inner retina (orange arrows). (b) mNPC expressed the indicator of presence of neuronal tissue after transplantation, anti-DAP (yellow arrows). The mNPC exhibited the neural-like morphology of living cells in the superficial retina. Donor cells occurred both as individual cells as well as occasional small clusters of cells associated with the host retina. (c) Living-cell marker, B-tubulin, enabled confirmation of donor cells in the inner retinal layers (white arrows), 40x.

 The experiments with this retinal degeneration model showed the survival, migration, and integration of neurospheres containing GFP-expressing mNPC (approximate 95% progenitor neural cells and 5% stem cells) up to 7 days using confocal microscopy. The clinically accessible GFP-marked neural cells were able to home to the injured retina, and the cellular agglomeration located within the inner layers of the retina. Detailed evaluation by the immunohistochemistry GFP-marked transplanted cells suggests their presence in the vicinity of the ganglion cell layer along the inner limiting membrane, although the distribution was not uniform (Figure 2). 

 The material analyzed with immunohistochemistry primarly revealed that the transplanted cells were “living” and of “neural origin” proved by antibodies anti-tubulin III and anti-DAP, respectively (Figure 2). The latter stains cells containing living nuclei, while the former would not color dead cells. The mNPC population in the transplants was tightly surrounded by neuroretinal cells, suggesting their active role in neuron survival. Notably, the appearance of GFP-positive mNPC was not the result of fusion between donor cells and endogenous neuroretinal cells. 

**Figure 3:**
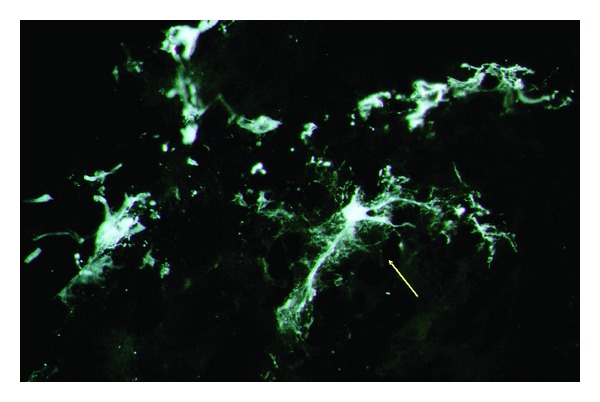
Few GPC-marker mNPC showed differentiation to mature neuronal cells, some of those cells showed prolongations resembling mature neurons by 1 week after injection. Cells altered from round cells to glia-like cells in a flat-cluster shape with an elongated bipolar shape oriented toward various directions (yellow arrow). 40x.

 Our investigation found that some mNPC eventually differentiated to mature neuronal cells, some of those cells showed prolongations resembling astrocytes or neurons. By 1 week after injection, grafted cells changed from round cells to glia-like cells in a flat cluster shape with an elongated bipolar shape oriented toward various directions (Figure 3). The level of differentiation of transplanted cells cannot be ascertained as GFP may induce false-positive results. In turn, in some areas of the inner retina the transplanted cells that migrated resembled undifferentiated precursors. 

 The architectural organization of the host retina was not disrupted by the transplantation procedure or cell migration. There was neither evidence of an immunologic response (e.g., invasion of macrophages or lymphocytes, edema) nor hemorrhage in any of the eyes included in this study.

## 4. Discussion

 Cell-based therapies for diseases such as retinitis pigmentosa have been restricted so far as a result of the minimal integration and differentiation of donor cells in the retinal architecture. In recent years, various studies showed a range of cell populations used for transplantation experiments in rodent retinas [2–5]. Specifically neural stem/progenitor cells isolated from central nervous system, the retina, or from embryonic stem cells consist in an attractive source for donor cells, as they expand in vitro and may differentiate into diverse neuronal cell types [2–4, 6, 7].

 Previous studies have shown conflicting results in regard to the migration and integration of intravitreal application neural progenitors into the retina. Johnson et al. as well as Canola and Arsenijevic observed little or no integration of intravitreal retinal stem cells into the host retina in mice [7, 8], as the inner membrane limitans appeared to be a barrier against incorporation of neural stem cells from the vitreous [9]. Significant retinal incorporation of transplanted cells apparently occurs only in certain conditions such as retinal injury or degeneration, enzyme digestion, cell-specific toxins, or transplanted before retinal development [2, 4, 6]. On the other hand, Young et al. showed widespread incorporation of adult rat hippocampal progenitor cells into the retina of dystrophic rats when injected intravitreally [4]. Herein, we presented that a subpopulation of NPCs isolated from telencephalon of embryonic mouse integrated the mice injured retina and survived for 7 days; the grafted NPCs cells preferentially integrated into ganglion cell and nerve fiber layer. 

 Stem/progenitor cells may serve as a cellular source to derive cell populations capable of differentiating into mature retinal cell type. So far, most reports demonstrated no specific differentiation of NPCs into retinal cells in laboratory studies. In contrast, few animal studies demonstrated astrocytes and photoreceptors differentiation after migration of mNPC after subretinal or intravitreal transplantation in the retina or central nervous system [9, 10]. In this study, we demonstrated that transplanted mNPC may differentiate into cells with elaborate neural morphologies resembling neurons or astrocytes in the retina. Such differentiation may have been facilitated by the absence of growth factors in the laboratory preparation. 

 In conclusion, migration, survival, and differentiation of mNPCs were observed after 7 days following a single application with neurosphere method. In the future, we aim to evaluate the recovery of retinal function; the cell type of differentiation; the inputs and extensions of axonal areas; and the comparison cell-based rescue therapies with different cellular types [11, 12].
